# Screening Based on Structural and Biological Verification of Stachyose as a PPARγ‐Modulating Ligand for the Treatment of Non‐Alcoholic Fatty Liver Disease

**DOI:** 10.1002/fsn3.71009

**Published:** 2025-09-22

**Authors:** Binbo Fang, Mengyuan Li, Feng Jiang, Weisong Dong, Weizhi Zhang, Lifan Lin, Yongheng Bai, Jianjian Zheng

**Affiliations:** ^1^ Zhejiang Key Laboratory of Intelligent Cancer Biomarker Discovery and Translation The First Affiliated Hospital of Wenzhou Medical University Wenzhou Zhejiang China; ^2^ School of Medicine Taizhou University Taizhou Zhejiang China

**Keywords:** acetylation, NAFLD, PPARγ, ROSI, stachyose

## Abstract

Peroxisome proliferator‐activated receptor gamma (PPARγ) is a critical therapeutic target for metabolic disorders like non‐alcoholic fatty liver disease (NAFLD). However, PPARγ full agonists such as rosiglitazone (ROSI) exhibit limited efficacy and off‐target effects. Intriguingly, transcriptomic analyses revealed dynamic PPARγ expression during NAFLD progression—compensatory upregulation in early stages and downregulation in advanced disease—highlighting the need for novel modulators. This study investigates the therapeutic potential of stachyose (STA), a natural bioactive compound, in NAFLD and its mechanism of action via PPARγ modulation. Using structure‐based virtual screening of 4531 natural compounds, STA was identified as a PPARγ‐targeted ligand, validated by surface plasmon resonance and molecular docking. Network pharmacology and functional enrichment analyses elucidated STA's multi‐target effects. In vitro and in vivo models assessed STA's impacts on lipid metabolism, inflammation, and insulin resistance. Molecular dynamics simulations and post‐translational modification studies clarified STA‐PPARγ interactions. STA outperformed ROSI in mitigating hepatic lipid accumulation, inflammation, and insulin resistance in both models. STA bound stably to PPARγ via residues GLU259, GLY284, PHE287, ILE341, and LEU270, with reduced PPARγ acetylation mediated by SIRT1 activation. Unlike ROSI, STA preserved PPARγ activity without inhibiting phosphorylation at Ser273, suggesting a distinct mechanism of action. STA emerges as a partial PPARγ agonist with superior efficacy and safety profiles compared to ROSI. Its dual role in enhancing fatty acid oxidation and suppressing lipogenesis, coupled with SIRT1‐dependent deacetylation of PPARγ, positions STA as a promising candidate for NAFLD therapy. This study provides a mechanistic foundation for developing PPARγ‐targeted interventions with reduced side effects.

## Graphical abstract

This study identifies stachyose (STA), a natural oligosaccharide, as a novel partial PPARγ agonist with superior therapeutic efficacy over rosiglitazone in non‐alcoholic fatty liver disease (NAFLD). STA uniquely stabilizes PPARγ through SIRT1‐mediated deacetylation while avoiding Ser273 phosphorylation, thereby enhancing fatty acid oxidation and suppressing lipogenesis without off‐target effects. These findings establish STA as a promising PPARγ‐targeted candidate for NAFLD therapy, offering a mechanistic blueprint for developing safer metabolic modulators.

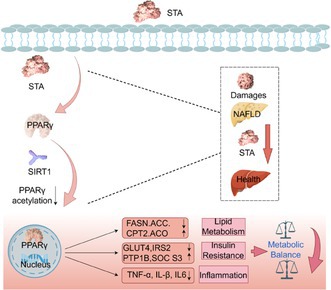

**Abbreviations**
ACC: acetyl‐CoA carboxylase
ACO2: aconitase 2
ALT: alanine aminotransferase
AST: aspartate aminotransferase
Co‐IP: co‐immunoprecipitation
FASN: fatty acid synthase
LPS: lipopolysaccharides
NAFLD: non‐alcoholic fatty liver disease
NASH: non‐alcoholic steatohepatitis
OA: oleic acid
ORO: Oil Red O Staining
PPARγ: peroxisome proliferator‐activated receptor γ
qRT‐PCR: quantitative real‐time PCR
Rg: radius of gyration
RMSD: root mean square deviation
RMSF: root mean square fluctuation
ROSI: rosiglitazone
SCD1: stearoyl‐CoA desaturase 1
SPR: surface plasmon resonance
STA: stachyose
TZD: thiazolidinedione
VLDL: very low‐density lipoprotein
WB: western blot

## 1 Background

In adult populations worldwide, NAFLD affects approximately 25% of the population, with its incidence increasing every year (Younossi et al. 2023). As NAFLD progresses, it can lead to non‐alcoholic steatohepatitis (NASH), a more serious condition that, if left untreated, may result in cirrhosis and cancer of the liver (Sheka et al. 2020). Pharmacological intervention becomes crucial at the NASH stage to prevent further disease progression. Given the serious consequences of disease progression, effective therapeutic strategies are urgently needed. However, as of the present time, only a single pharmaceutical agent has received clinical approval for the treatment of NASH (Kingwell 2024). In light of this, it underscores the necessity of conducting further research into NAFLD pathogenesis in order to identify novel diagnostic and therapeutic targets.

PPARs (α, β, γ) are key transcription factors that regulate inflammation and disease progression (Madariaga Traconis et al. 2024; Wang et al. 2020). As key regulators of inflammation and fatty acid oxidation, PPARs are attractive targets for NASH treatments (Boeckmans et al. 2019; Gross et al. 2017). Among these, PPARγ has emerged as one of the most promising targets for treating metabolic diseases such as NASH. Activation of PPARγ has been shown to ameliorate NASH in preclinical models by reducing hepatic inflammation, oxidative stress, and insulin resistance (Huang et al. 2025; Li et al. 2022; Singh et al. 2024). Due to the PPAR structure, several thiazolidinedione (TZD) drugs have been developed to treat type 2 diabetes, such as rosiglitazone (ROSI) (Dang et al. 2021). Despite their therapeutic advantages, thiazolidinedione (TZD) drugs are linked to significant adverse effects, including weight gain, fluid retention, myocardial hypertrophy, and hepatotoxicity, which constrain their clinical utility (Carrasco et al. 2023). The broad activation of downstream genes by full PPARγ agonists contributes to these adverse effects, highlighting the need for new PPARγ‐based therapies with more selective mechanisms of action (Ma et al. 2021).

STA is a naturally occurring carbohydrate found in various plants and traditional Chinese medicines, including coptis, soybeans, and beets. It has been reported to have blood glucose‐lowering and anti‐inflammatory properties, as well as protective effects against liver and biliary diseases (Cao et al. 2020; Li et al. 2021; Ren et al. 2024). For instance, STA has been shown to reduce malondialdehyde and protein carbonyl levels, while enhancing glutathione peroxidase and superoxide dismutase activities, leading to decreased liver injury markers in high‐fructose‐fed mice (Li et al. 2016). These studies suggest that STA has potential anti‐inflammatory, hypoglycemic, and hepatoprotective effects, but its role in NAFLD has not been fully elucidated.

The principal aim of this study was to examine the therapeutic efficacy of STA in the treatment of NAFLD and to elucidate the role of the PPARγ signaling pathway in mediating the effects of STA. Our findings demonstrate that STA, acting as a partial agonist of PPARγ, markedly decreased hepatic lipid accumulation, inflammation, and insulin resistance, with greater efficacy than the full agonist, ROSI. Specifically, STA directly binds to PPARγ, promoting its deacetylation and thereby effectively impeding the progression of NAFLD. These findings indicate that STA has potential as a natural therapeutic agent for the treatment of NAFLD.

## 2 Materials and Methods

### 2.1 Materials

STA and ROSI were procured from MedChemExpress. Oleic acid (OA) and Lipopolysaccharides (LPS) were sourced from Sigma (Cat# O1008 and Cat# LPS25).

### 2.2 Cell Culture and Treatment

The HepG2 human liver cancer cells were purchased from Wuhan Pricella Biotechnology Co. Ltd. (Catalog number: CL‐0103) and were cultured in complete Dulbecco's Modified Eagle Medium (DMEM) supplemented with 10% fetal bovine serum (FBS) and 1% penicillin–streptomycin antibiotic solution. Model establishment: The cells were first cultured in the corresponding serum‐free medium for a duration of 12 h. Subsequently, they were incubated with 0.4 mM OA and 2 μg/mL LPS for an additional 48 h (Linghu et al. 2023).

### 2.3 Animal Experiments

For our experiments, we used eight‐week‐old male C57BL/6 mice as controls. All mice were maintained under a 12‐h light–dark cycle with unrestricted access to food and water. NAFLD was induced by administering a high‐fat diet (D12492; Research Diets; protein 20%; fat 60%; carbohydrate 20%; Beijing, China) to the mice for a duration of 24 weeks. The STA and ROSI doses were prepared as follows: STA was dissolved in a vehicle (blank solution) and administered to the mice via daily gavage at two concentrations, 300 mg/kg and 600 mg/kg. ROSI was dissolved in the same vehicle and gavaged at a dose of 5 mg/kg. The control group was treated with the vehicle (blank solution) only, without STA or ROSI administration. The animal studies conducted at Taizhou University have received approval from the Institutional Animal Ethics Committee.

### 2.4 Target Prediction for STA and NAFLD

For the analysis of STA's 2D and 3D structures, PubChem (https://pubchem.ncbi.nlm.nih.gov/) was used in SDF format. Use the SwissTargetPrediction to search for the action targets of STA. Targets related to NAFLD were retrieved from GeneCards, OMIM, Therapeutic Target Database (TTD), and PHARMGKB. A Venn diagram of overlapping genes was created using Sangerbox.

### 2.5 GO and KEGG Analysis

Functional enrichment analysis was conducted using the KEGG REST API and R package clusterProfiler (v3.14.3). GO annotation was performed using org.Hs.eg.db (v3.1.0). For statistical significance, we used *p* < 0.05 and FDR < 0.1, with a minimum gene set of 5 and a maximum gene set of 5000.

### 2.6 CCK‐8 Assay

Cells in the exponential growth phase were seeded in a 96‐well plate at 5.0 × 10^3^ cells per well and incubated. Afterward, 10 μL of CCK‐8 solution was added to each well and incubated for 2 h. Cell viability was measured by reading the optical density at 450 nm with a microplate reader.

### 2.7 Cell Oil Red O Staining (ORO)

Fix the cells with 4% paraformaldehyde for 20 min, soak in 60% isopropanol, stain with Oil Red O (G1262; Solarbio, China) for 30 min, and capture images using an optical microscope.

### 2.8 Histological Analysis

After the organization of sample collection is completed, subsequently perform H&E, Bodipy, and ORO staining on paraffin‐embedded and frozen liver sections according to the established protocol (Jiang et al. 2025). Subsequently, microscopic images of the tissue sections were obtained.

### 2.9 Quantitative Real‐Time PCR (qRT‐PCR)

RNA was extracted using Trizol reagent (Thermo Fisher Scientific, Catalog number: 15596026). cDNA was synthesized with a TaKaRa PrimeScript RT Reagent Kit (TaKaRa, Catalog number: RR037A). qRT‐PCR was performed on an Applied Biosystems 7500 Fast Real‐Time PCR System (Applied Biosystems, USA) using TaKaRa SYBR Premix Ex Taq II (TaKaRa, Catalog number: RR820A), with primers listed in Table S1.

### 2.10 Biochemical Index Testing

Cellular biochemical indices included intracellular TG. For this measurement, cells were seeded in 6‐well plates at a density of 1 × 10^6^ cells per well and cultured under appropriate conditions. After reaching approximately 80% confluence, the cells were washed twice with cold PBS to remove any residual media and then harvested for TG content analysis using a Triglyceride Colorimetric Assay Kit. In addition, blood glucose, AST, ALT levels in mouse serum, and TG content in mouse liver tissue were measured. For serum collection, mice were fasted for 6 h before blood was collected via tail vein or cardiac puncture. The blood was then centrifuged at 4°C for 10 min at 3000 rpm to separate the serum, which was stored at −80°C until further analysis. Blood glucose was measured using a Glucose Assay Kit (Catalog number: K606‐100, BioVision, USA), while AST and ALT levels were measured using AST and ALT Activity Assay Kits (Catalog numbers: MAK055 and MAK052, Sigma‐Aldrich, USA). For liver tissue, approximately 50–100 mg of liver was collected from each mouse, weighed, and homogenized in cold PBS. The tissue homogenate was centrifuged at 4°C, and the supernatant was used for triglyceride content measurement using the Triglyceride Assay Kit (Catalog number: 10010303, Abcam, UK). All procedures were performed according to the manufacturer's protocols for the respective kits, and results were expressed relative to tissue weight or serum volume.

### 2.11 Western Blot (WB)

Protein concentration was measured with a BCA Protein Assay Kit (Catalog number: 23227, Thermo Fisher Scientific, USA) after sample lysis. WB analyses were performed with anti‐acetyl‐CoA carboxylase (ACC; A15606, ABclonal), anti‐TNFα (A11534, ABclonal), anti‐IRS2 (A7945, ABclonal), anti‐Acetyl‐Lys (ab22550, ab190479, Abcam), anti‐SIRT1 (ab189494, ab110304, Abcam), anti‐GAPDH (A19056, ABclonal), anti‐PPARγ (ab316981, ab41928, Abcam), and anti‐pSer273 PPARγ (bs4888R, Bioss) antibodies.

### 2.12 Virtual Screening

The virtual screening workflow module facilitated the screening process by importing the prepared compounds and performing molecular docking through the Glide module, which aligns the geometries and energies of the receptor and ligand molecules. Initially, the standard mode of the Glide module was used for preliminary screening of small synthetic compounds. Subsequently, the top 15% of compounds were selected for further evaluation using the high‐precision mode to determine the ranking of the small synthetic compounds.

### 2.13 Surface Plasmon Resonance (SPR)

We utilize the amine coupling method for protein immobilization, using 1.0 × PBS‐P+ (pH 7.4) as the protein coupling buffer and 1.0 × PBS‐P+ (pH 7.4) containing 5% (v/v) DMSO as the interaction buffer. After placing the running buffer, water, and waste containers in their respective instrument trays, a CM5 chip is inserted into the slot. Channel 2 of the chip is activated using EDC and NHS, and the ligand protein, diluted to 50 μg/mL, is immobilized on this channel, followed by blocking with ethanolamine. Channel 1 serves as a reference, undergoing the same procedure without protein immobilization. For the interaction analysis between Human PPARG protein and test compounds, a solvent correction is first performed using a 5% DMSO concentration calibration curve. Test compounds are serially diluted in a 96‐well plate and sequentially coupled with the target protein from low to high concentrations at a flow rate of 30 μL/min for 150 s. After each test, the chip is regenerated for 5 min using 10 mM glycine‐HCl solution (pH 2.0). Data are collected using BIAcore T200 Control software and fitted to a 1:1 Langmuir binding model using BIAcore T200 Evaluation software to obtain binding and dissociation constants.

### 2.14 Molecular Docking Analysis

Molecular docking was conducted employing Autodock software, with the active site enclosed within the docking box and a docking duration of 100 units. Standard settings were applied for all other variables.

### 2.15 Molecular Dynamics Simulation and Analysis

A 200 ns molecular dynamics simulation using Gromacs software examined the interaction between STA and PPARγ. The reference conformation was based on the predicted docking structure. The Root Mean Square Deviation (RMSD), Radius of Gyration (Rg), and Root Mean Square Fluctuation (RMSF) values of the complex were calculated using gmx rms, gmx gyrate, and gmx rmsf commands, respectively.

### 2.16 Virtual Amino Acid Mutation Analysis

The conformation with the lowest energy obtained from the molecular dynamic simulation was chosen, and the mutation energy of the complex was determined using the ASM analysis in Discovery Studio 2021 software to assess the significance of specific amino acid residues in the binding of STA to PPARγ.

### 2.17 Co‐Immunoprecipitation (Co‐IP)

After protein extraction and quantification, a small portion was reserved as an input control. The remaining protein was split and incubated with either primary antibodies (anti‐acetyl‐lysine or anti‐SIRT1) or IgG antibody (negative control). For PPARγ acetylation analysis, anti‐PPARγ antibody was used for immunoprecipitation, followed by Western blot with anti‐acetyl‐lysine antibody. To confirm SIRT1‐mediated PPARγ deacetylation, the same immunoprecipitation was performed, but Western blot used anti‐SIRT1 antibody. The resulting supernatants were analyzed by Western blot to assess STA's effect on PPARγ acetylation and SIRT1's role in this process.

### 2.18 Statistical Analysis

Results are shown as the mean ± SD. To measure the difference between two groups or two groups at different time points, nonparametric either two‐tailed t test or two‐way ANOVA was employed by the GraphPad Prism software package. *p* < 0.05 was considered statistically significant.

## 3 Results

### 3.1 Screening of Natural Compounds Based on PPARγ

**FIGURE 1:**
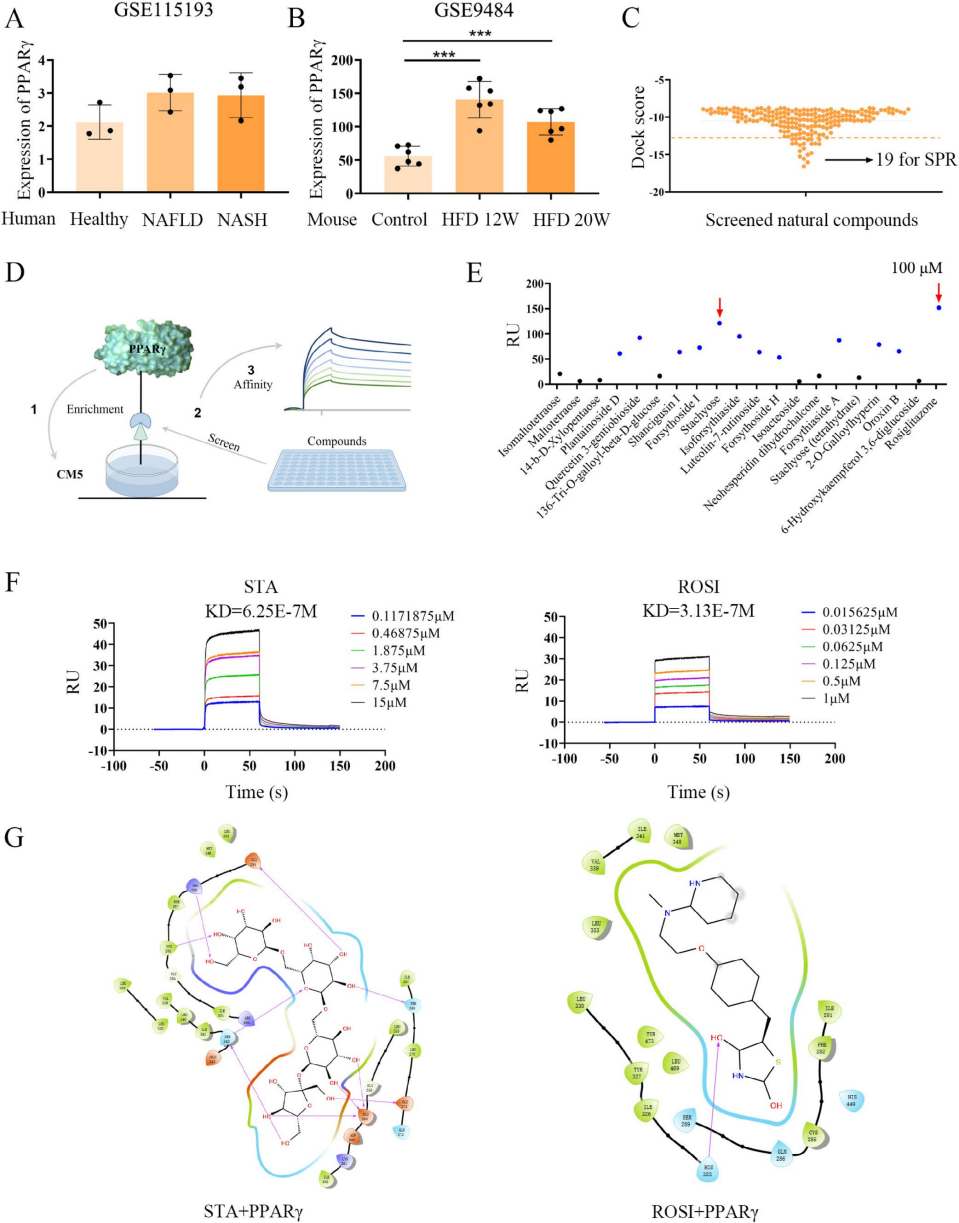
STA binds stably with PPARγ. (A) PPARγ expression in NAFLD patients. (B) PPARγ expression in HFD mice. (C) Utilizing virtual screening techniques to identify natural compounds that target PPARγ. (D) Schematic diagram of SPR. (E) SPR single concentration response values for screening compounds. (F) SPR multi‐concentration response values for STA and ROSI. (G) The 2D docking structures of STA and ROSI with PPARγ, respectively. ***p < 0.001.

Many studies have shown that PPARγ plays a role in NAFLD development. Consistent with these findings, analysis of GEO transcriptomic sequencing datasets (accession numbers: GSE9484, GSE115193) revealed that PPARγ was compensatorily upregulated in early‐stage NAFLD but downregulated in late‐stage disease (Figure 1A,B) (Qiu et al. 2023). To identify potential natural compounds that could modulate PPARγ and impact NAFLD progression, we performed virtual screening based on the three‐dimensional structure of PPARγ (PDB ID: 1ZGY). The HY‐L021 Natural Compound Library is a high‐quality screening library developed by MedChemExpress (MCE), widely used in drug discovery, chemical biology research, and related fields. From the HY‐L021 natural product library containing 4531 natural compounds, we identified 200 potential PPARγ ligands (Figure 1C). Subsequently, the top 19 compounds selected based on docking scores, along with the known PPARγ agonist ROSI, were subjected to SPR analysis for validation (Figure 1D). Despite the strict filtering criteria used in the virtual screening process, SPR results indicated some false positives among the selected compounds. Therefore, STA, which exhibited a response unit value closest to ROSI, was chosen for multi‐concentration SPR analysis (Figure 1E). The dissociation constant (KD) values for STA and ROSI were 6.25E‐7 M and 3.13E‐7 M, respectively, indicating that STA is a promising candidate as a targeted PPARγ ligand (Figure 1F). Molecular docking further confirmed that STA shares structural similarities with ROSI in its interaction with PPARγ (Figure 1G). In summary, we identified a potential targeted PPARγ protein compound, STA, through a combination of virtual screening and affinity screening.

### 3.2 Exploring the Therapeutic Potential of STA for NAFLD: A Network Pharmacology Approach

**FIGURE 2:**
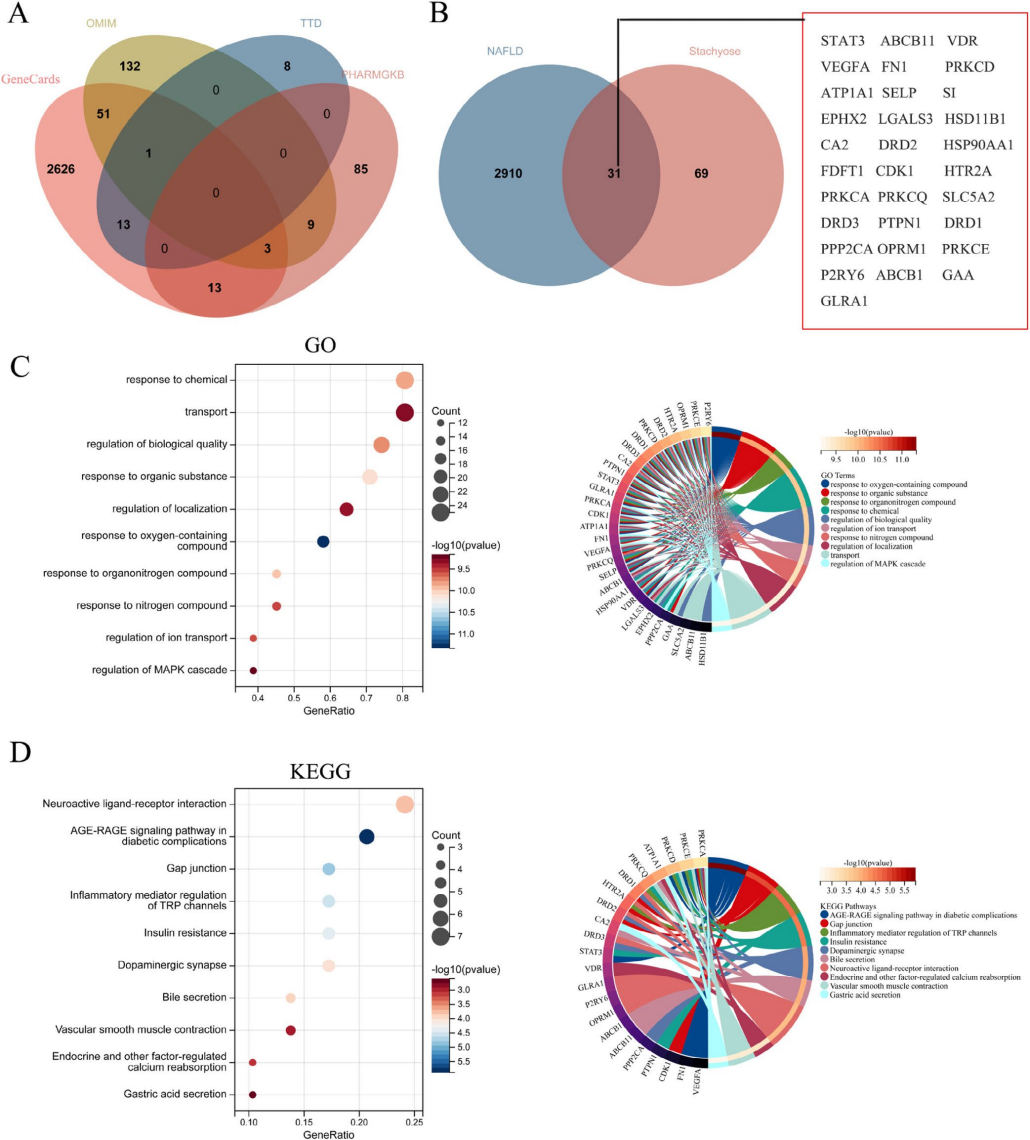
Therapeutic potential of STA in NAFLD. (A) Targets for NAFLD from four databases. (B) Common targets between NAFLD and STA. (C) GO functional enrichment analysis of 31 intersecting genes. (D) KEGG functional enrichment analysis of 31 intersecting genes.

We identified STA, a natural product that acts on PPARγ, through virtual screening and SPR validation. Subsequently, we performed a network pharmacology analysis to explore STA's therapeutic potential for NAFLD. Disease‐related genes for NAFLD were collected from the GeneCards, OMIM, TTD, and PHARMGKB databases (Figure 2A), and STA's potential targets were identified using the SwissTargetPrediction database. We found 31 common targets between STA and NAFLD (Figure 2B). We then conducted GO and KEGG functional enrichment analyses on these 31 genes. In the GO analysis, the genes were enriched in the following biological processes: response to chemical, transport, regulation of biological quality, response to organic substance, regulation of localization, response to oxygen‐containing compound, response to organonitrogen compound, response to nitrogen compound, regulation of ion transport, and regulation of MAPK cascade (Figure 2C). In the KEGG analysis, the genes were enriched in pathways including neuroactive ligand‐receptor interaction, AGE‐RAGE signaling pathway in diabetic complications, gap junction, inflammatory mediator regulation of TRP channels, insulin resistance, dopaminergic synapse, bile secretion, vascular smooth muscle contraction, endocrine and other factor‐regulated calcium reabsorption, and gastric acid secretion (Figure 2D). These findings suggest that STA has therapeutic potential for NAFLD.

### 3.3 STA Alleviates Lipid Generation, Inflammation, and Insulin Resistance In Vitro

**FIGURE 3:**
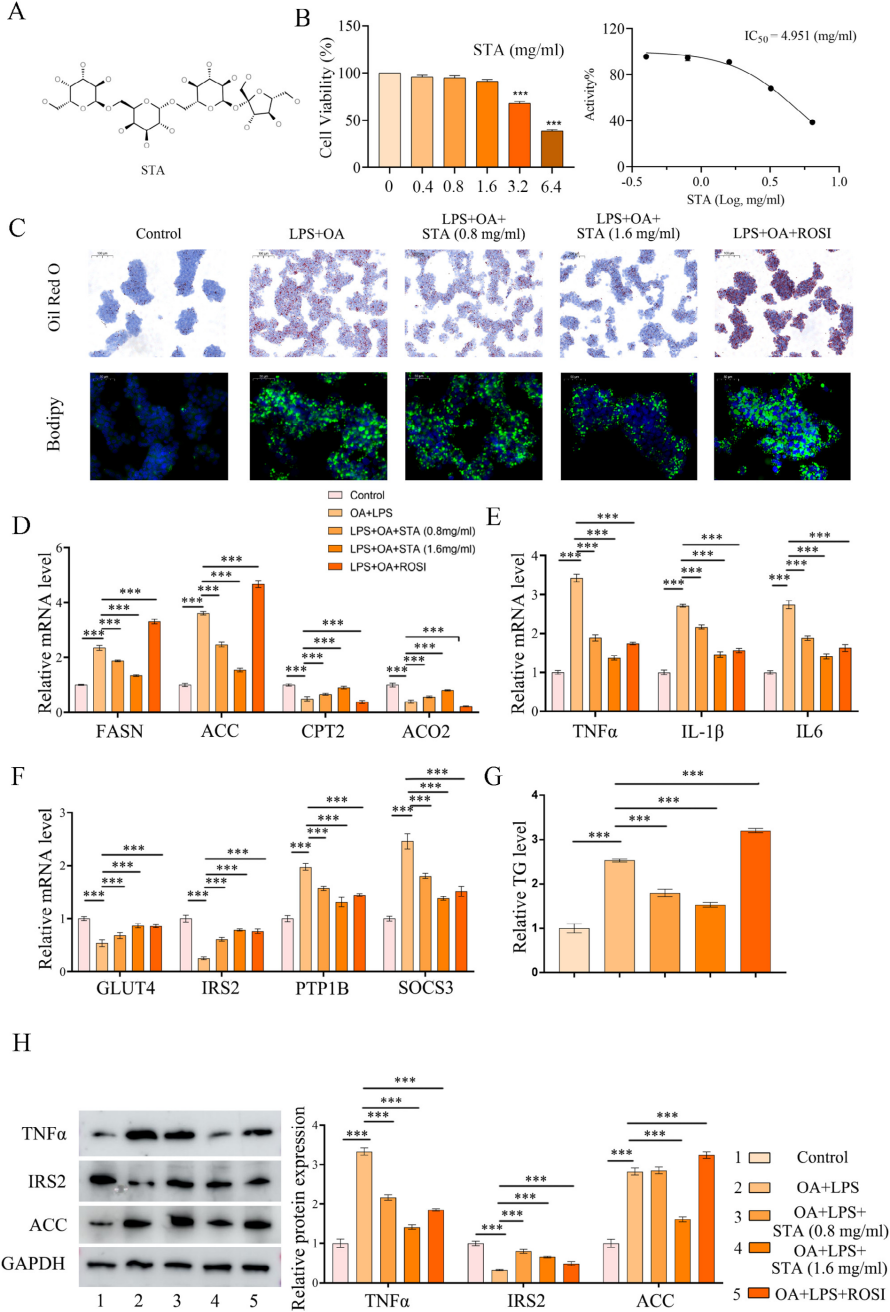
STA improves lipid generation, inflammation, and insulin resistance in a model of hepatic lipid accumulation. (A) Chemical structure of STA. (B) Cytotoxicity of STA on HepG2 cells. (C) ORO staining images of HepG2 cells. (D) qRT‐PCR results of lipid metabolism‐related molecules (FASN, ACC, CPT2, ACO2) in HepG2 cells. (E) qRT‐PCR results of inflammation‐related molecules (TNF‐α, IL‐1β, IL6) in HepG2 cells. (F) qRT‐PCR results of insulin resistance‐related molecules (GLUT4, IRS2, PTP1B, SOCS3) in HepG2 cells. (G) Detection of TG content in HepG2 cells. (H) Protein expression of TNFα, IRS2, and ACC in HepG2 cells. ***p < 0.001.

HepG2 cells treated with OA and LPS for 24 h were used to model lipid overaccumulation. STA, a natural small alkaloid found in various plants and traditional Chinese medicines, was evaluated for its effects on these cells (Figure 3A). Cell viability analysis showed that STA's toxicity increased with concentration, leading us to use 0.8 and 1.6 mg/mL STA for subsequent experiments (Figure 3B). Compared to STA, ROSI significantly promoted lipid droplet accumulation based on TG content and ORO staining (Figure 3C,G). At the mRNA level, STA upregulated genes involved in fatty acid oxidation (CPT2 and AO2) and downregulated genes associated with lipogenesis (FASN and ACC), demonstrating superior lipid‐lowering effects compared to ROSI (Figure 3D). STA additionally attenuated the expression of inflammatory markers, including IL‐6, IL‐1β, and TNFα (Figure 3E). Furthermore, it mitigated insulin resistance by upregulating the expression of insulin‐sensitive factors, such as GLUT4 and IRS2, while concurrently downregulating insulin resistance factors, including PTP1B and SOCS3 (Figure 3F). Protein‐level analyses corroborated these findings (Figure 3H). These findings suggested that the therapeutic efficacy of STA in the hepatocyte lipid deposition model surpassed that of ROSI.

### 3.4 STA Ameliorates Hepatic Steatosis, Inflammation, and Insulin Resistance in HFD Mice

**FIGURE 4:**
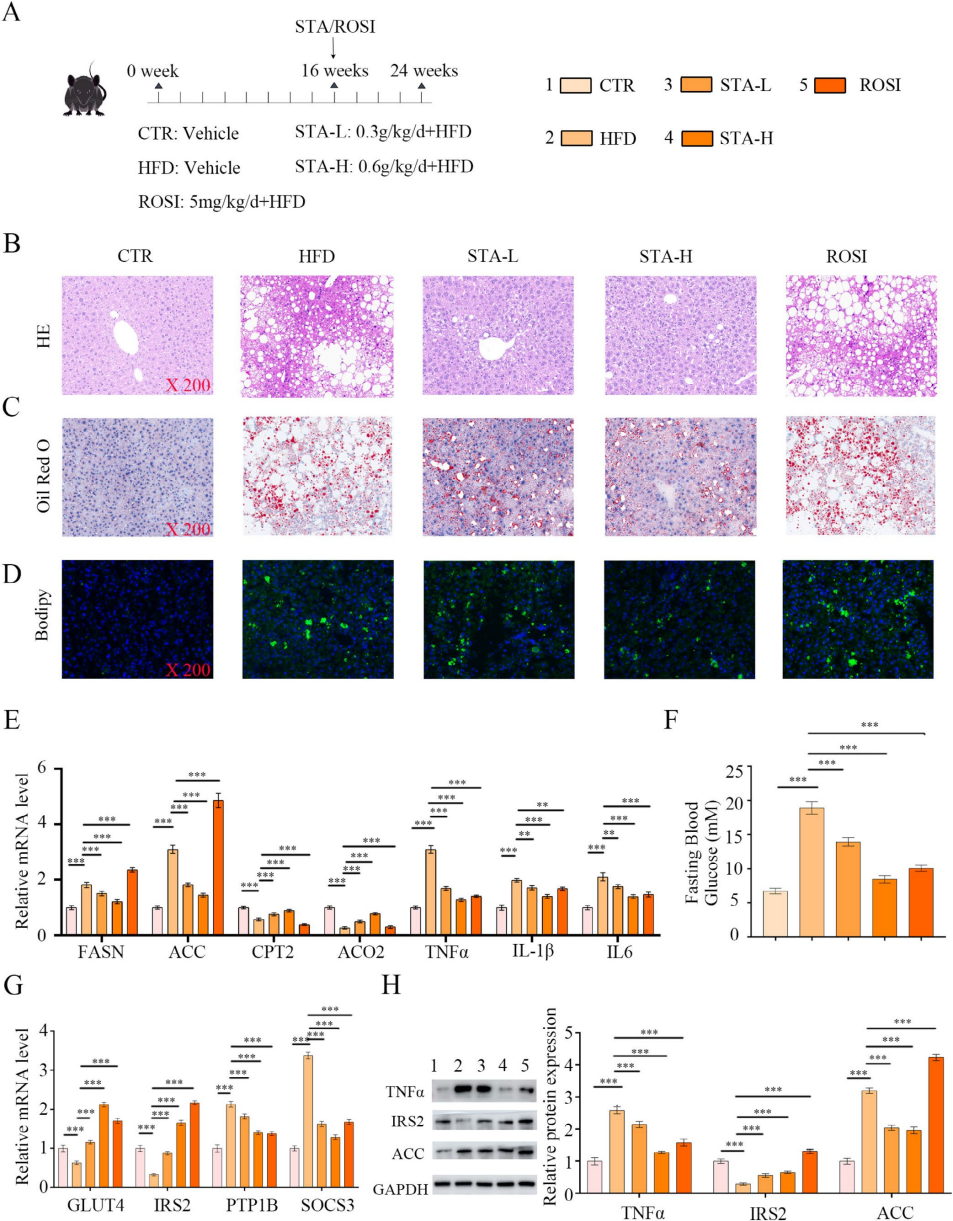
STA improves lipid metabolism, inflammation, and insulin resistance induced by HFD in mice. (A) Experimental steps of STA on HFD‐induced mice, *n* = 6 in each group. (B) H&E staining images. (C) ORO staining images. (D) Bodipy staining images. (E) qRT‐PCR results of lipid metabolism‐related molecules (FASN, ACC, CPT2, ACO2) and inflammation‐related molecules (TNF‐α, IL‐1β, IL6) in mice liver tissues. (F) Detection of fasting blood glucose. (G) qRT‐PCR results of insulin resistance‐related molecules (GLUT4, IRS2, PTP1B, SOCS3) in mice liver tissues. (H) Protein expression of ACC, TNFα, and IRS2 in mice liver tissues. ***p < 0.001.

The therapeutic efficacy of STA in ameliorating hepatic steatosis, inflammation, and insulin resistance was evaluated in murine models subjected to a high‐fat diet (Figure 4A). Histological analyses using HE, ORO, and Bodipy revealed that STA significantly reduced hepatocyte injury and lipid accumulation in HFD‐fed mice (Figure 4B–D, Figure S1A–D). Long‐term HFD feeding impaired the liver's capacity to clear TG and accumulated lipids, a condition that STA effectively improved. STA treatment downregulated lipogenic genes (FASN and ACC) and upregulated genes related to fatty acid oxidation (CPT2 and ACO2) in the liver (Figure 4E). In alignment with the in vitro findings, STA significantly reduced the levels of inflammatory cytokines, including TNF‐ɑ, IL‐1β, and IL‐6 (Figure 4E). Furthermore, STA ameliorated insulin resistance, as evidenced by the upregulation of GLUT4 and IRS2 expression and the downregulation of PTP1B and SOCS3 expression (Figure 4G). STA also lowered fasting blood glucose levels (Figure 4F). The protein‐level analyses supported the gene expression findings (Figure 4H). In summary, STA treatment confers protection against obesity, inflammation, insulin resistance, and hepatic dysfunction induced by a high‐fat diet.

### 3.5 Stable Binding of STA to PPARγ and Its Binding Sites

**FIGURE 5:**
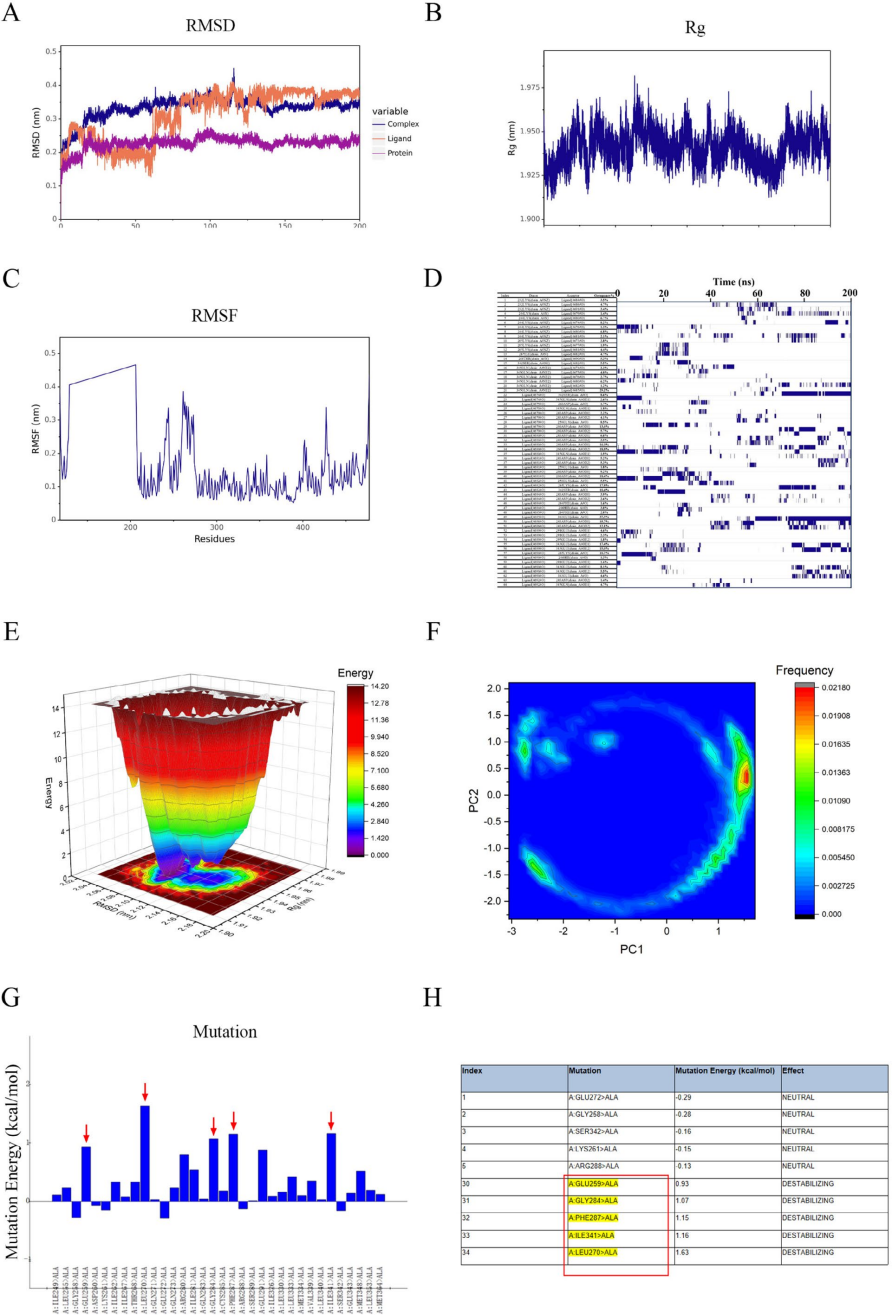
Key amino acid residues involved in the interaction between STA and PPARγ. (A) RMSD of the complex, protein, and small molecule ligand. (B) Rg of the complex. (C) RMSF of the protein within the complex. (D) Hydrogen Bond Frequency Analysis between PPARγ and STA. (E) Free Energy Landscape between PPARγ and STA. (F) Principal Component Analysis between PPARγ and STA. (G, H) ASM analysis of PPARγ LBD and STA.

To further explore the interaction between STA and PPARγ, molecular dynamics simulations and alanine scanning mutagenesis were performed. As shown in Figure 5A, the RMSD of the STA and PPARγ complex structure gradually stabilized as the simulation progressed, indicating that the complex structure becomes increasingly stable over time. Figure 5B demonstrated that the Rg (radius of gyration) of the complex remained essentially stable throughout the simulation, further confirming the structural stability of the complex. The RMSF (Root Mean Square Fluctuation) results revealed the presence of highly flexible amino acids surrounding STA, suggesting that the binding conformation of STA is susceptible to adjustments (Figure 5C). Figure 5D indicated that the hydrogen bonds between STA and PPARγ exhibited relatively low stability. The complex existed in a single low‐energy state, implying the overall structural stability of the complex (Figure 5E). As illustrated in Figure 5F, STA possessed a complex structure, making it prone to significant conformational changes. In the PCA (Principal Component Analysis) plot, the conformations of the small molecule were widely distributed, with one high‐frequency conformation standing out (Figure 5F). These findings collectively provide insights into the dynamic behavior and stability of the STA‐PPARγ complex, highlighting areas of flexibility and conformational changes that may be relevant to its function or interactions. Alanine scanning mutagenesis identified key residues, GLU259, GLY284, PHE287, ILE341, and LEU270, critical for the STA‐PPARγ interaction (Figure 5G,H). These results confirm that STA stably binds to PPARγ, with specific residues playing significant roles in this interaction.

### 3.6 STA Promotes the Deacetylation of PPARγ to Improve NAFLD

**FIGURE 6:**
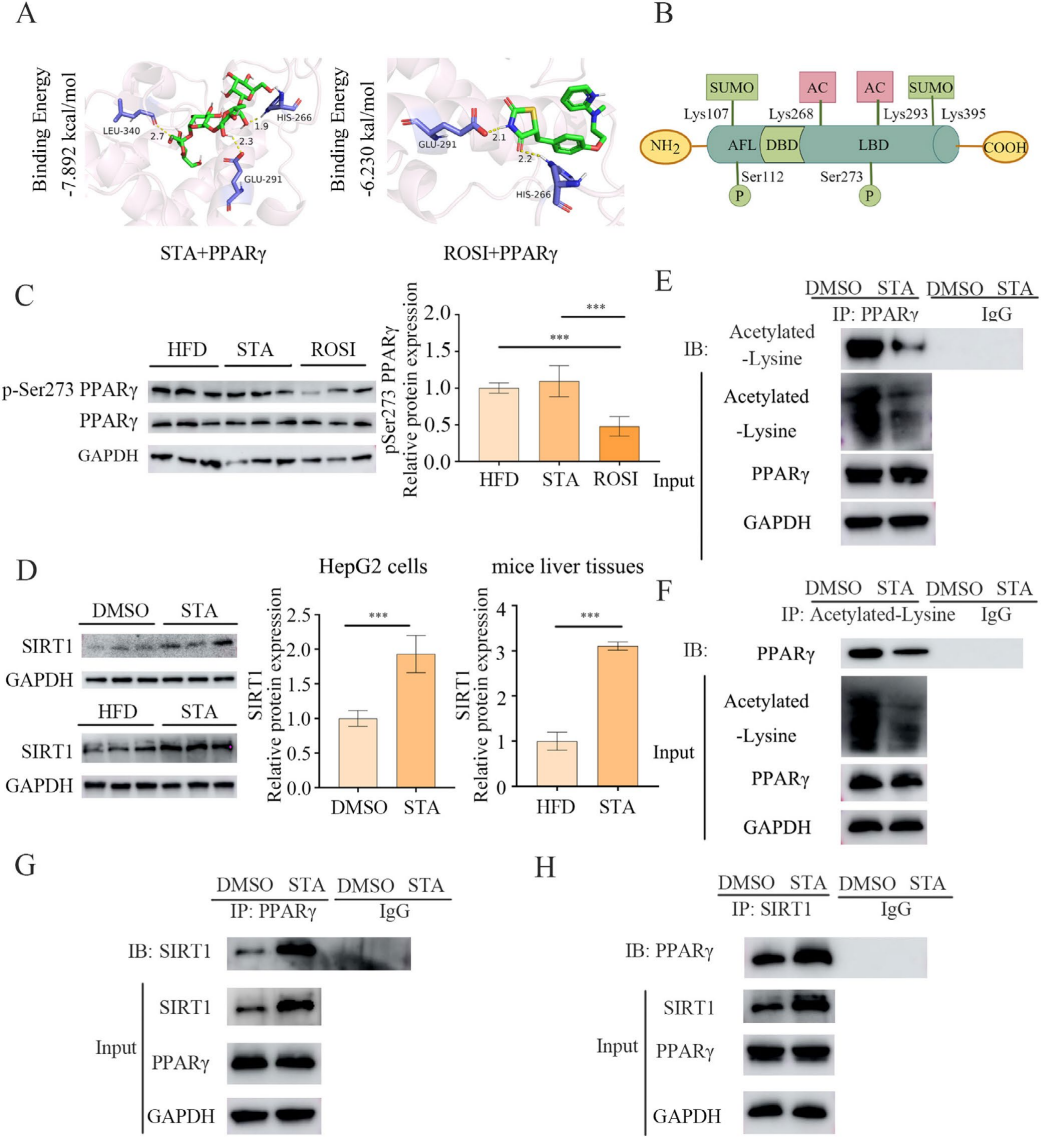
STA regulates the phosphorylation and acetylation of PPARγ. (A) Molecular Docking Diagram. (B) Schematic diagram of post‐translational modification sites of PPARγ. (C) Protein expression of pSer273 PPARγ and PPARγ in mice liver tissues. (D) Protein expression of SIRT1 in mice liver tissues and HepG2 cells. (E, F) Co‐IP analysis of the interaction between SIRT1 and PPARγ in HepG2 cells. (G, H) Co‐IP analysis of PPARγ deacetylation in HepG2 cells. ***p < 0.001.

The molecular mechanism underlying STA's effects on NAFLD was further investigated. Molecular docking suggested stable binding of STA to PPARγ, with binding energies (−7.892 kcal/mol), similar to those of ROSI (Figure 6A). Given that STA does not significantly alter PPARγ expression, we speculate that its proximity to acetylation and phosphorylation sites may allow STA to influence PPARγ's post–translational modifications, a direction we intend to explore in our research (Figure 6B). The observation that STA did not significantly affect Ser273 phosphorylation of PPARγ, similar to the HFD‐fed group, suggests this site is not the specific target of STA (Figure 6C), consistent with other studies (Linghu et al. 2023; Ma et al. 2021). Instead, our focus shifted to acetylation. Notably, SIRT1, a critical deacetylase involved in the regulation of PPARγ deacetylation, exerts its effects both in vivo and in vitro, as illustrated in Figure 6D. ROSI had no significant effect on the expression level of SIRT1 (Figure S2). Co‐IP analysis demonstrated that STA markedly reduced PPARγ acetylation levels (Figure 6E,F). Further Co‐IP assays confirmed the involvement of SIRT1 in STA‐induced PPARγ deacetylation (Figure 6G,H). Collectively, the data indicate that STA improves NAFLD through SIRT1‐dependent deacetylation of PPARγ.

## 4 Discussion

The increasing prevalence and economic impact of NAFLD have established it as a significant public health concern. PPARγ is key in NAFLD development, affecting lipid buildup, insulin resistance, inflammation, oxidative and ER stress, and fibrosis, making it a potential treatment target (Chen et al. 2023; Qiu et al. 2023). Our study shows that STA, a natural compound, effectively slows NAFLD progression in vitro and in vivo. Notably, STA was found to directly bind to PPARγ, promoting its deacetylation. Collectively, STA shows potential as a potential therapeutic intervention for NAFLD.

The initial stage in the progression of NAFLD is characterized by lipid accumulation within hepatocytes (Li et al. 2025). *De novo* lipogenesis in the liver denotes the biochemical conversion of non‐fatty acid substrates, such as glucose, lactate, and amino acids, into fatty acids. This process is facilitated by key enzymatic reactions involving ACC, FASN, and stearoyl‐CoA desaturase 1 (SCD1) (Softic et al. 2016). The metabolism of fatty acids predominantly occurs through β‐oxidation within mitochondria and peroxisomes (Kersten and Stienstra 2017). In this study, STA demonstrated lipid‐lowering effects in hepatocytes stimulated with OA and LPS, consistent with its lipid‐reducing action observed in HFD‐fed mice. This effect showed significant improvement compared to the potent adipogenic activity of the full PPARγ agonist ROSI, both in vitro and in vivo.

Hepatic inflammation is a hallmark of advancing NAFLD (Peiseler et al. 2022). Sustained liver damage in NAFLD often leads to chronic inflammation, which exacerbates fibrosis and can result in liver fibrotic lesions (Weiskirchen et al. 2019). Studies have shown that long‐term consumption of STA can improve colon and liver inflammation associated with HFD and its complications by modulating the gut microbiota (Liu et al. 2019). Our study found that STA effectively suppressed the expressions of proinflammatory cytokines TNFα, IL6, and IL1β, indicating its strong anti‐inflammatory properties. Insulin resistance plays a pivotal role in the pathogenesis of NAFLD by impairing hepatic lipid metabolism processes such as *de novo* lipogenesis, β‐oxidation, and the synthesis of very low‐density lipoprotein (VLDL) (Palma et al. 2022). Elevated free fatty acids can lead to significant lipid accumulation and oxidative damage in the liver, promoting steatosis (Geng et al. 2021; Zhang et al. 2024). Insulin resistance significantly affects glucose and lipid metabolism, leading to increased breakdown of dysfunctional fat tissue and decreased glucose uptake in muscles (Sasaki et al. 2025). Consistent with the previous findings, STA upregulated key factors associated with insulin sensitivity, such as GLUT4 and IRS2, while downregulating insulin resistance factors like PTP1B and SOCS3 in both cell cultures and animal models. Moreover, the administration of STA led to a significant decrease in blood glucose levels.

Researchers are increasingly concentrating on the development of PPARγ modulators exhibiting partial agonist activity. This approach aims to preserve the therapeutic advantages of mitigating insulin resistance, inflammation, and oxidative stress, while concurrently minimizing the adverse effects associated with obesity (Ma et al. 2022). Prior studies have demonstrated that PPARγ is subject to a range of post‐translational modifications, encompassing acetylation, phosphorylation, and ubiquitination (Carvalho et al. 2021; Quan et al. 2019). Emerging evidence suggests that PPARγ deacetylation can selectively induce browning of adipose tissue without adverse effects (Xu et al. 2021). Deacetylation appears to achieve the goal of separating the metabolic benefits of PPARγ activation from its adverse effects. Utilizing a strategy of PPARγ deacetylation may lead to the design of safer and more effective agonists of this nuclear receptor for the treatment of metabolic diseases (Kraakman et al. 2018). Furthermore, compounds that inhibit cdk5‐mediated PPARγ Ser273 phosphorylation have shown promise in reducing insulin resistance (Dias et al. 2020; Haas et al. 2024). However, our study found that STA‐induced PPARγ dephosphorylation was similar to that in the model group, suggesting that STA's anti‐NAFLD effect is not achieved through PPARγ dephosphorylation. Instead, the acetylation of PPARγ, particularly near the STA binding, became the focus of our study. SIRT1, a NAD^+^‐dependent protein deacetylase known for its role in cellular metabolic regulation, can deacetylate PPARγ in a ligand‐specific manner (Yu et al. 2024). Consistent with prior research, STA treatment resulted in the upregulation of SIRT1, thereby promoting the deacetylation of PPARγ. This mechanistic pathway may elucidate the observed differences in efficacy between STA and ROSI.

**FIGURE 7:**
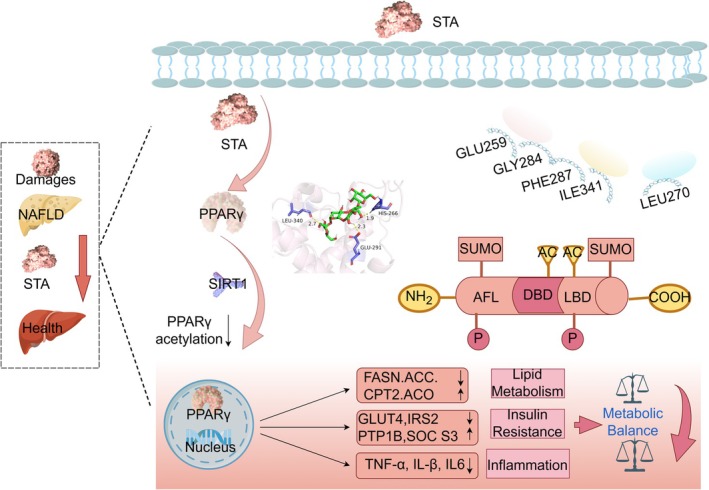
Molecular mechanism of STA targeting PPARγ for the treatment of NAFLD. STA has been proven to directly bind to the PPARγ protein, promoting the deacetylation of PPARγ.

Natural products have long served as a reservoir of pharmaceutical compounds, with many modern drugs derived from natural sources, often rooted in traditional medicine (El‐Seedi et al. 2025; Najmi et al. 2022). As drug development advances, the demand for screening chemical diversity has increased, and natural products will continue to play a key role in meeting this demand (Domingo‐Fernández et al. 2024; Guo et al. 2022). To identify potent, natural, and low‐toxic compounds, we performed virtual screening based on the PPARγ structure. STA emerged as the most promising candidate for further investigation in NAFLD models. Molecular docking analysis revealed that STA binds favorably to PPARγ, with a binding site distinct from that of the PPARγ full agonist ROSI. Molecular dynamic simulations further suggested stable binding between STA and PPARγ. Using the ASM method, we identified critical residues, GLU259, GLY284, PHE287, ILE341, and LEU270, that significantly impact STA binding to PPARγ. Collectively, STA appears to stably bind to PPARγ at these sites and shows potential as a PPARγ ligand for managing NAFLD. This expands the possibilities for utilizing a natural product in NAFLD treatment and targeting PPARγ in NAFLD therapy (Figure 7).

## 5 Conclusions

This study establishes STA as a prototype of second‐generation PPARγ modulators that combine ligand‐binding selectivity with epigenetic fine‐tuning. By leveraging endogenous SIRT1 activity to restore PPARγ function in advanced NAFLD, STA circumvents the “all‐or‐none” limitations of classical agonists. The STA‐PPARγ‐SIRT1 axis represents a feed‐forward loop connecting nutrient availability to transcriptional regulation—a mechanism that may generalize to other nuclear receptor‐targeted therapies. Our integrative approach, from computational screening to post‐translational modification mapping, provides a blueprint for developing context‐sensitive metabolic therapeutics.

## Author Contributions

**Binbo Fang:** data curation (lead), investigation (lead), methodology (lead), project administration (lead), resources (lead), software (lead), writing – original draft (lead). **Mengyuan Li:** data curation (equal), formal analysis (lead), investigation (equal), methodology (equal), writing – original draft (equal). **Feng Jiang:** data curation (equal), writing – original draft (equal). **Weisong Dong:** data curation (equal), methodology (equal), writing – original draft (equal). **Weizhi Zhang:** data curation (equal), formal analysis (equal), writing – original draft (equal). **Lifan Lin:** data curation (equal), formal analysis (equal). **Yongheng Bai:** supervision (equal), writing – review and editing (equal). **Jianjian Zheng:** conceptualization (lead), supervision (lead), writing – review and editing (equal).

## Ethics Statement

The Experimental Animal Welfare and Ethics Committee of Taizhou University approved reporting this case.

## Conflicts of Interest

The authors declare no conflicts of interest.

## Supporting information


**Figure S1:** Role of STA in the HFD Model.
**Figure S2:** Regulation of SIRT1 by ROSI.
**Table S1:** The primer sequences.

## Data Availability

Data from this study is available upon reasonable request to the corresponding author.

## References

[fsn371009-bib-0001] Boeckmans, J. , A. Natale , M. Rombaut , et al. 2019. “Anti‐NASH Drug Development Hitches a Lift on PPAR Agonism.” Cells 9: 37. 31877771 10.3390/cells9010037 PMC7016963

[fsn371009-bib-0002] Cao, H. , C. Li , L. Lei , et al. 2020. “Stachyose Improves the Effects of Berberine on Glucose Metabolism by Regulating Intestinal Microbiota and Short‐Chain Fatty Acids in Spontaneous Type 2 Diabetic KKAy Mice.” Frontiers in Pharmacology 11: 578943. 33192521 10.3389/fphar.2020.578943 PMC7642818

[fsn371009-bib-0003] Carrasco, A. G. , A. Izquierdo‐Lahuerta , A. M. Valverde , et al. 2023. “The Protective Role of Peroxisome Proliferator‐Activated Receptor Gamma in Lipotoxic Podocytes.” Biochimica et Biophysica Acta—Molecular and Cell Biology of Lipids 1868: 159329. 37156296 10.1016/j.bbalip.2023.159329

[fsn371009-bib-0004] Carvalho, M. V. , C. F. Gonçalves‐de‐Albuquerque , and A. R. Silva . 2021. “PPAR Gamma: From Definition to Molecular Targets and Therapy of Lung Diseases.” International Journal of Molecular Sciences 22: 805. 33467433 10.3390/ijms22020805 PMC7830538

[fsn371009-bib-0005] Chen, H. , H. Tan , J. Wan , et al. 2023. “PPAR‐γ Signaling in Nonalcoholic Fatty Liver Disease: Pathogenesis and Therapeutic Targets.” Pharmacology & Therapeutics 245: 108391. 36963510 10.1016/j.pharmthera.2023.108391

[fsn371009-bib-0006] Dang, Y. F. , S. H. Yang , X. N. Jiang , et al. 2021. “Combination Treatment Strategies With a Focus on Rosiglitazone and Adriamycin for Insulin Resistant Liver Cancer.” Journal of Drug Targeting 29: 336–348. 33115283 10.1080/1061186X.2020.1844216

[fsn371009-bib-0007] Dias, M. M. G. , F. A. H. Batista , T. H. Tittanegro , et al. 2020. “PPARγ S273 Phosphorylation Modifies the Dynamics of Coregulator Proteins Recruitment.” Frontiers in Endocrinology 11: 561256. 33329381 10.3389/fendo.2020.561256 PMC7729135

[fsn371009-bib-0008] Domingo‐Fernández, D. , Y. Gadiya , A. J. Preto , et al. 2024. “Natural Products Have Increased Rates of Clinical Trial Success Throughout the Drug Development Process.” Journal of Natural Products 87: 1844–1851. 38970498 10.1021/acs.jnatprod.4c00581 PMC11287737

[fsn371009-bib-0009] El‐Seedi, H. R. , M. S. Refaey , N. Elias , et al. 2025. “Marine Natural Products as a Source of Novel Anticancer Drugs: An Updated Review (2019‐2023).” Natural Products and Bioprospecting 15: 13. 39853457 10.1007/s13659-024-00493-5 PMC11759743

[fsn371009-bib-0010] Geng, Y. , K. N. Faber , V. E. de Meijer , H. Blokzijl , and H. Moshage . 2021. “How Does Hepatic Lipid Accumulation Lead to Lipotoxicity in Non‐Alcoholic Fatty Liver Disease?” Hepatology International 15: 21–35. 33548031 10.1007/s12072-020-10121-2 PMC7886759

[fsn371009-bib-0011] Gross, B. , M. Pawlak , P. Lefebvre , and B. Staels . 2017. “PPARs in Obesity‐Induced T2DM, Dyslipidaemia and NAFLD.” Nature Reviews. Endocrinology 13: 36–49. 10.1038/nrendo.2016.135 27636730

[fsn371009-bib-0012] Guo, X. , X. Yin , Z. Liu , and J. Wang . 2022. “Non‐Alcoholic Fatty Liver Disease (NAFLD) Pathogenesis and Natural Products for Prevention and Treatment.” International Journal of Molecular Sciences 23: 15489. 36555127 10.3390/ijms232415489 PMC9779435

[fsn371009-bib-0013] Haas, B. , M. D. S. Hass , A. Voltz , et al. 2024. “Sulfonylureas Exert Antidiabetic Action on Adipocytes by Inhibition of PPARγ Serine 273 Phosphorylation.” Molecular Metabolism 85: 101956. 38735390 10.1016/j.molmet.2024.101956 PMC11112612

[fsn371009-bib-0014] Huang, C. C. , C. H. Wang , H. Y. Yeh , et al. 2025. “Peroxisome Proliferator‐Activated Receptor α/γ and Cannabinoid Receptor 2 Agonist Attenuated Nonalcoholic Steatohepatitis Exosome‐Related Abnormalities in Mice.” American Journal of Pathology 195: 188–203. 39490440 10.1016/j.ajpath.2024.10.006 PMC12179500

[fsn371009-bib-0015] Jiang, F. , X. Li , L. Lin , M. Li , and J. Zheng . 2025. “NPRC Promotes Hepatic Steatosis via USP30‐Mediated Deubiquitination of C/EBPβ.” Metabolism 162: 156050. 39433172 10.1016/j.metabol.2024.156050

[fsn371009-bib-0016] Kersten, S. , and R. Stienstra . 2017. “The Role and Regulation of the Peroxisome Proliferator Activated Receptor Alpha in Human Liver.” Biochimie 136: 75–84. 28077274 10.1016/j.biochi.2016.12.019

[fsn371009-bib-0017] Kingwell, K. 2024. “NASH Field Celebrates ‘hurrah Moment’ With a First FDA Drug Approval for the Liver Disease.” Nature Reviews. Drug Discovery 23: 235–237. 10.1038/d41573-024-00051-1 38486072

[fsn371009-bib-0018] Kraakman, M. J. , Q. Liu , J. Postigo‐Fernandez , et al. 2018. “PPARγ Deacetylation Dissociates Thiazolidinedione's Metabolic Benefits From Its Adverse Effects.” Journal of Clinical Investigation 128: 2600–2612. 29589839 10.1172/JCI98709 PMC5983311

[fsn371009-bib-0019] Li, C. , H. Cao , Y. Huan , et al. 2021. “Berberine Combined With Stachyose Improves Glycometabolism and Gut Microbiota Through Regulating Colonic microRNA and Gene Expression in Diabetic Rats.” Life Sciences 284: 119928. 34480937 10.1016/j.lfs.2021.119928

[fsn371009-bib-0020] Li, F. , J. Peng , H. Feng , et al. 2022. “KLF9 Aggravates Streptozotocin‐Induced Diabetic Cardiomyopathy by Inhibiting PPARγ/NRF2 Signalling.” Cells 11: 3393. 36359788 10.3390/cells11213393 PMC9656075

[fsn371009-bib-0021] Li, L. , Z. Guo , Y. Zhao , et al. 2025. “The Impact of Oxidative Stress on Abnormal Lipid Metabolism‐Mediated Disease Development.” Archives of Biochemistry and Biophysics 766: 110348. 39961502 10.1016/j.abb.2025.110348

[fsn371009-bib-0022] Li, W. , Z. Li , X. Han , D. Huang , Y. Lu , and X. Yang . 2016. “Enhancing the Hepatic Protective Effect of Genistein by Oral Administration With Stachyose in Mice With Chronic High Fructose Diet Consumption.” Food & Function 7: 2420–2430. 27157892 10.1039/c6fo00038j

[fsn371009-bib-0023] Linghu, L. , W. Zong , Y. Liao , et al. 2023. “Herpetrione, a New Type of PPARα Ligand as a Therapeutic Strategy Against Nonalcoholic Steatohepatitis.” Research 6: 0276. 38034083 10.34133/research.0276 PMC10687582

[fsn371009-bib-0024] Liu, Y. , T. Li , A. Alim , D. Ren , Y. Zhao , and X. Yang . 2019. “Regulatory Effects of Stachyose on Colonic and Hepatic Inflammation, Gut Microbiota Dysbiosis, and Peripheral CD4(+) T Cell Distribution Abnormality in High‐Fat Diet‐Fed Mice.” Journal of Agricultural and Food Chemistry 67: 11665–11674. 31588753 10.1021/acs.jafc.9b04731

[fsn371009-bib-0025] Ma, L. , Y. Lian , J. Tang , et al. 2021. “Identification of the Anti‐Fungal Drug Fenticonazole Nitrate as a Novel PPARγ‐Modulating Ligand With Good Therapeutic Index: Structure‐Based Screening and Biological Validation.” Pharmacological Research 173: 105860. 34461220 10.1016/j.phrs.2021.105860

[fsn371009-bib-0026] Ma, L. , J. Tang , G. Cai , et al. 2022. “Structure‐Based Screening and Biological Validation of the Anti‐Thrombotic Drug‐Dicoumarol as a Novel and Potent PPARγ‐Modulating Ligand.” Bioorganic Chemistry 129: 106191. 36270169 10.1016/j.bioorg.2022.106191

[fsn371009-bib-0027] Madariaga Traconis, A. P. , M. Uribe‐Esquivel , and V. J. Barbero Becerra . 2024. “Exploring the Role of Peroxisome Proliferator‐Activated Receptors and Endothelial Dysfunction in Metabolic Dysfunction‐Associated Steatotic Liver Disease.” Cells 13: 2055. 39768147 10.3390/cells13242055 PMC11674254

[fsn371009-bib-0028] Najmi, A. , S. A. Javed , M. Al Bratty , and H. A. Alhazmi . 2022. “Modern Approaches in the Discovery and Development of Plant‐Based Natural Products and Their Analogues as Potential Therapeutic Agents.” Molecules 27: 349. 35056662 10.3390/molecules27020349 PMC8779633

[fsn371009-bib-0029] Palma, R. , A. Pronio , M. Romeo , et al. 2022. “The Role of Insulin Resistance in Fueling NAFLD Pathogenesis: From Molecular Mechanisms to Clinical Implications.” Journal of Clinical Medicine 11: 3649. 35806934 10.3390/jcm11133649 PMC9267803

[fsn371009-bib-0030] Peiseler, M. , R. Schwabe , J. Hampe , P. Kubes , M. Heikenwälder , and F. Tacke . 2022. “Immune Mechanisms Linking Metabolic Injury to Inflammation and Fibrosis in Fatty Liver Disease—Novel Insights Into Cellular Communication Circuits.” Journal of Hepatology 77: 1136–1160. 35750137 10.1016/j.jhep.2022.06.012

[fsn371009-bib-0031] Qiu, Y. Y. , J. Zhang , F. Y. Zeng , and Y. Z. Zhu . 2023. “Roles of the Peroxisome Proliferator‐Activated Receptors (PPARs) in the Pathogenesis of Nonalcoholic Fatty Liver Disease (NAFLD).” Pharmacological Research 192: 106786. 37146924 10.1016/j.phrs.2023.106786

[fsn371009-bib-0032] Quan, Q. , Y. Qian , X. Li , and M. Li . 2019. “Pioglitazone Reduces β Amyloid Levels via Inhibition of PPARγ Phosphorylation in a Neuronal Model of Alzheimer's Disease.” Frontiers in Aging Neuroscience 11: 178. 31379559 10.3389/fnagi.2019.00178 PMC6650543

[fsn371009-bib-0033] Ren, D. , M. Ding , J. Su , et al. 2024. “Stachyose in Combination With *L. rhamnosus* GG Ameliorates Acute Hypobaric Hypoxia‐Induced Intestinal Barrier Dysfunction Through Alleviating Inflammatory Response and Oxidative Stress.” Free Radical Biology & Medicine 212: 505–519. 38211833 10.1016/j.freeradbiomed.2024.01.009

[fsn371009-bib-0034] Sasaki, N. , Y. Ueno , R. Ozono , Y. Nakano , and Y. Higashi . 2025. “Insulin Resistance in Adipose Tissue and Fatty Liver, but Not Fat Mass, Are Involved in Worsening Glycaemic Status: The Hiroshima Study on Glucose Metabolism and Cardiovascular Diseases.” Diabetes, Obesity & Metabolism 27: 3025–3035. 10.1111/dom.16307 40045548

[fsn371009-bib-0035] Sheka, A. C. , O. Adeyi , J. Thompson , B. Hameed , P. A. Crawford , and S. Ikramuddin . 2020. “Nonalcoholic Steatohepatitis: A Review.” JAMA 323: 1175–1183. 32207804 10.1001/jama.2020.2298

[fsn371009-bib-0036] Singh, S. , A. Kumar , S. Gupta , and R. Agrawal . 2024. “Curative Role of Natural PPARγ Agonist in Non‐Alcoholic Fatty Liver Disease (NAFLD).” Tissue Barriers 12: 2289830. 38050958 10.1080/21688370.2023.2289830 PMC11262216

[fsn371009-bib-0037] Softic, S. , D. E. Cohen , and C. R. Kahn . 2016. “Role of Dietary Fructose and Hepatic De Novo Lipogenesis in Fatty Liver Disease.” Digestive Diseases and Sciences 61: 1282–1293. 26856717 10.1007/s10620-016-4054-0 PMC4838515

[fsn371009-bib-0038] Wang, Y. , T. Nakajima , F. J. Gonzalez , and N. Tanaka . 2020. “PPARs as Metabolic Regulators in the Liver: Lessons From Liver‐Specific PPAR‐Null Mice.” International Journal of Molecular Sciences 21: 2061. 32192216 10.3390/ijms21062061 PMC7139552

[fsn371009-bib-0039] Weiskirchen, R. , S. Weiskirchen , and F. Tacke . 2019. “Organ and Tissue Fibrosis: Molecular Signals, Cellular Mechanisms and Translational Implications.” Molecular Aspects of Medicine 65: 2–15. 29958900 10.1016/j.mam.2018.06.003

[fsn371009-bib-0040] Xu, Y. , T. Yu , G. Ma , et al. 2021. “Berberine Modulates Deacetylation of PPARγ to Promote Adipose Tissue Remodeling and Thermogenesis via AMPK/SIRT1 Pathway.” International Journal of Biological Sciences 17: 3173–3187. 34421358 10.7150/ijbs.62556 PMC8375237

[fsn371009-bib-0041] Younossi, Z. M. , G. Wong , Q. M. Anstee , and L. Henry . 2023. “The Global Burden of Liver Disease.” Clinical Gastroenterology and Hepatology 21: 1978–1991. 37121527 10.1016/j.cgh.2023.04.015

[fsn371009-bib-0042] Yu, A. , R. Yu , H. Liu , C. Ge , and W. Dang . 2024. “SIRT1 Safeguards Adipogenic Differentiation by Orchestrating Anti‐Oxidative Responses and Suppressing Cellular Senescence.” Geroscience 46: 1107–1127. 37420111 10.1007/s11357-023-00863-w PMC10828476

[fsn371009-bib-0043] Zhang, J. , H. Ouyang , X. Gu , et al. 2024. “Caffeic Acid Ameliorates Metabolic Dysfunction‐Associated Steatotic Liver Disease via Alleviating Oxidative Damage and Lipid Accumulation in Hepatocytes Through Activating Nrf2 via Targeting Keap1.” Free Radical Biology & Medicine 224: 352–365. 39209138 10.1016/j.freeradbiomed.2024.08.038

