# Supplementary material for: Screening Based on Structural and Biological Verification of Stachyose as a PPARγ‐Modulating Ligand for the Treatment of Non‐Alcoholic Fatty Liver Disease
Source: Food Sci Nutr. 2025 Sep 22;13(9):e71009. doi: 10.1002/fsn3.71009 (PMC12454680; doi:10.1002/fsn3.71009)
Supplement: Supplementary file 1 — Figure S1: Role of STA in the HFD Model. Figure S2: Regulation of SIRT1 by ROSI. Table S1: The primer sequences. [file FSN3-13-e71009-s001.docx]

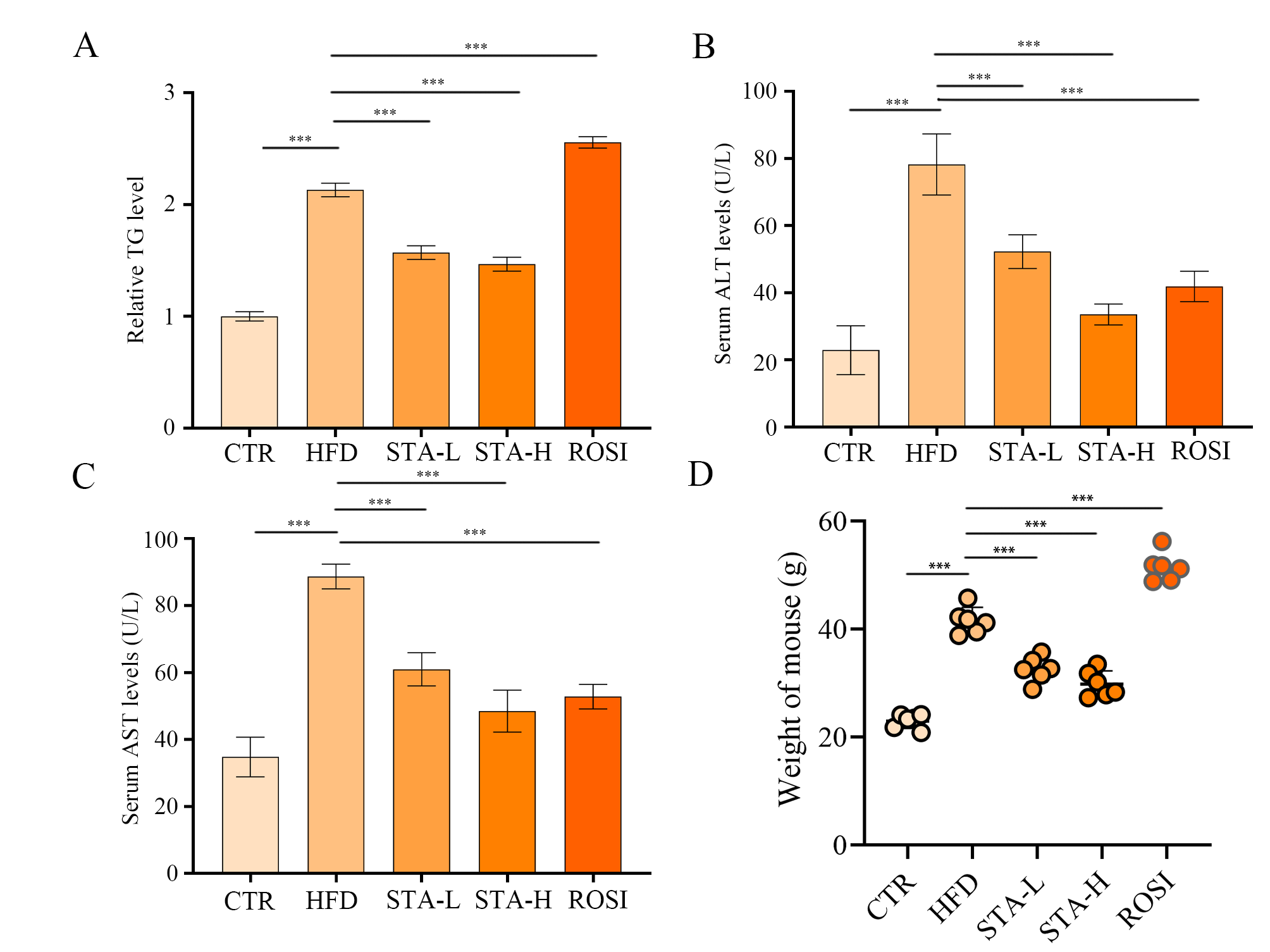


**Figure S1. Role of STA in the HFD Model.**

(A) Detection of TG content in mice in each group. (B) Concentration of serum ALT in mice. (C) Concentration of serum AST in mice. (D) After 8 weeks of treatment, the weight of the mice in each group was recorded. *** p < 0.001.

**
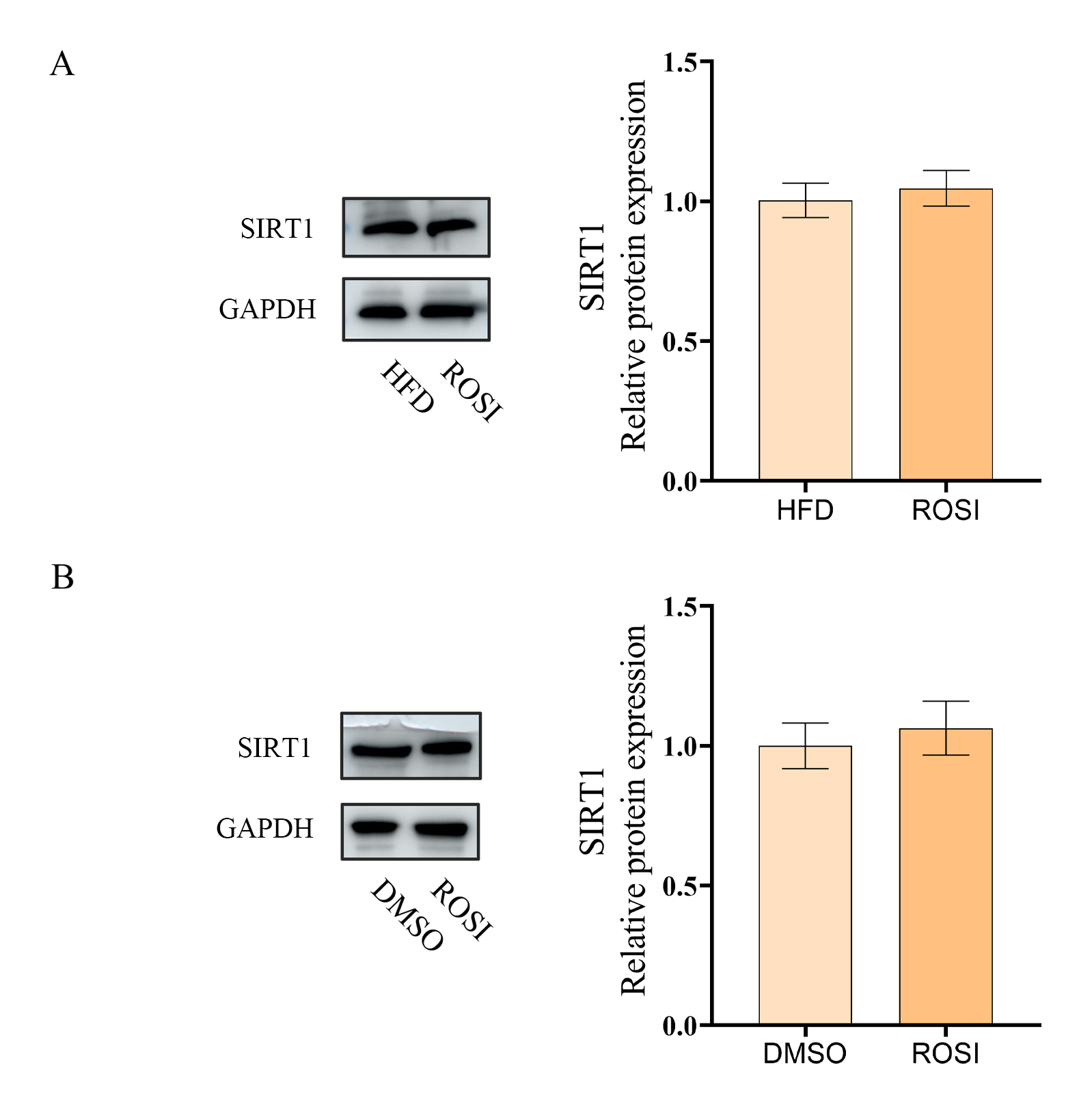
**

**Figure S2. Regulation of SIRT1 by ROSI.**

1. Protein expression of SIRT1 in mice liver tissues. (B) Protein expression of SIRT1 in HepG2 cells.

**Table S1: The primer sequences**

| Gene | Primers |
| --- | --- |
| FASN (mouse) | F1: ACAGCGGGGAATGGGTACT |
|  | R1:GCTTGGTCCTTTGAAGTCGAAGA |
| ACC (mouse) | F1: TCCGTCAGCTCAGATACAC |
|  | R1:GACATGCTGGATCTCATGTG |
| CPT2 (mouse) | F1: CCTGCTCGCTCAGGATAAACA |
|  | R1:GTGTCTTCAGAAACCGCACTG |
| ACO2 (mouse) | F1: AACCGGCCTCTTACTCTCTCA |
|  | R1:CAGGTATGTCTTTCCCCGCTC |
| TNFɑ (mouse) | F1: GGTGCCTATGTCTCAGCCTC |
|  | R1:CAGATTGACCTCAGCGCTGA |
| IL-1β (mouse) | F1: GTGGCAGCTACCTGTGTCTT |
|  | R1:CTCTGCTTGTGAGGTGCTGA |
| IL6 (mouse) | F1: TCTATACCACTTCACAAGTCGGA |
|  | R1:GAATTGCCATTGCACAACTCTTT |
| GLUT4 (mouse) | F1: AACACTCAACCAACTGGCCA |
|  | R1:CACCGAGACCAACGTGAAGA |
| IRS2 (mouse) | F1: ACCGACTTGGTCAGCGAAG |
|  | R1:CACGAGCCCGTAGTTGTCAT |
| PTP1B (mouse) | F1: CATTCGACATGAAGCCAGCG |
|  | R1:TGAGGAAAGAAGCCGGTGAC |
| SOCS3 (mouse) | F1: GCGGGCACCTTTCTTATCC |
|  | R1:CTGGAGGCGGCATGTAGTG |
| GAPDH (mouse) | F1: TTCACCACCATGGAGAAGGC |
|  | R1: TGAAGTCGCAGGAGACAACC |
| FASN (human) | F1: ACCTCCGTGCAGTTCTTGAG |
|  | R1: TTGGGGCTATGGAAGTGCAG |
| ACC (human) | F1: GGAGGAGGAGGGAAGGGAAT |
|  | R1: CGAGCAGCAATAACATGGCC |
| CPT2 (human) | F1: CCGTCCACTTTGAGCACTCT |
|  | R1: GCCATGGTACTTGGAGCACT |
| ACO2 (human) | F1: ATCTGGGTGGCATTGTCCTG |
|  | R1:GGCCTTGCCGTTTTCAATGT |
| TNFɑ (human) | F1: GTGCTTGTTCCTCAGCCTCT |
|  | R1: CACCCTTCTCCAGCTGGAAG |
| IL-1β (human) | F1: CCAGCTACGAATCTCCGACC |
|  | R1: GGGAACTGGGCAGACTCAAA |
| IL6 (human) | F1: TTCGGTCCAGTTGCCTTCTC |
|  | R1: AGCTGCGCAGAATGAGATGA |
| GLUT4 (human) | F1: CATCCAGAATCTCGAGGGGC |
|  | R1: GTTGCTCGTCCAGTTGGAGA |
| IRS2 (human) | F1: AGGACCTACTCCCTGACCAC |
|  | R1: GTTGAGGTAGTCCCCGTTGG |
| PTP1B (human) | F1: CTGCCTCTTGCTGATGGACA |
|  | R1: AACTCAGTGCATGGTCCTCG |
| SOCS3 (human) | F1: TGGTCACCCACAGCAAGTTT |
|  | R1: AGATGCTGAAGAGTGGCCAC |
| GAPDH (human) | F1: GAGAAGGCTGGGGCTCATTT |
|  | R1: GTCAAAGGTGGAGGAGTGGG |
